# Working towards Culturally Responsive Trauma-Informed Care in the Refugee Resettlement Process: Qualitative Inquiry with Refugee-Serving Professionals in the United States

**DOI:** 10.3390/bs11110155

**Published:** 2021-11-07

**Authors:** Hyojin Im, Laura E. T. Swan

**Affiliations:** 1 School of Social Work, Virginia Commonwealth University, 1000 Floyd Ave., 3rd Floor, Richmond, VA 23284, USA; 2 Department of Population Health Sciences, University of Wisconsin, Madison, WI 53726, USA; lswan2@wisc.edu

**Keywords:** trauma-informed care (TIC), refugee resettlement, culturally responsive care, human services, mental health and psychosocial support (MHPSS)

## Abstract

Trauma-informed care (TIC) approaches have gained popularity in various contexts of human services over the past decades. However, relatively little has been explored about how it is applicable and built into services for refugee populations in resettlement programs. This study explores the current status of the application of TIC in refugee-serving agencies and identifies perceived and experienced challenges and opportunities for culturally responsive TIC in the United States. As designed as part of the evaluation of state-wide refugee health promotion programs, this study conducted individual interviews with 78 refugee service providers from five resettlement sites. Despite the burgeoning interest and attempt to embrace TIC, our findings show that there is clear inconsistency and inexperience in TIC adaptation in resettlement programs. This study highlights that TIC that is culturally responsive and relevant to refugee trauma and acculturation experiences is a vital way to address the chasms between refugee-specific programs and mainstream services including mental health care systems. This study also discusses community resources and opportunities to bridge the deep divide and substantial gaps between mental health services and refugee resettlement services and to address comprehensive needs around mental health and wellness in the refugee community.

## 1. Introduction

Trauma-informed or trauma-sensitive approaches have gained popularity in various contexts of human services over the past decades [1,2]. Specifically, trauma-informed care frameworks have been adopted and implemented in various service settings including but not limited to mental and behavioral services [3], public healthcare [4], juvenile correctional systems and child welfare [5], schools [6], homeless shelters [7], and various programs serving survivors of violence [8,9]. Trauma-informed care (TIC) is defined by the Substance Abuse and Mental Health Services Administration (SAMHSA) [10] as follows:

A program, organization, or system that is trauma-informed realizes the widespread impact of trauma and understands potential paths for recovery; recognizes the signs and symptoms of trauma in clients, families, staff, and others involved with the system; and responds by fully integrating knowledge about trauma into policies, procedures, and practices, and seeks to actively resist re-traumatization.

(p. 9)

TIC is considered as: (1) an organizational or care system’s culture and policy-backed structure; and (2) interventions and direct practice approaches [11] that encompass several common elements or principles. As per SAMHSA [10], six key principles of TIC should be in place to ensure a care system or organization is sensitive and responsive to the trauma-related issues of their clients and staff. These principles include: (1) safety; (2) trustworthiness and transparency; (3) peer support; (4) collaboration and mutuality; (5) empowerment, voice, and choice; and (6) cultural, historical, and gender issues. TIC is also commensurate with the inclusion and involvement of service consumers, such as clients and their family members and caregivers. Collaborations between providers and consumers transpire throughout service planning and delivery in consumer-centered TIC [12].

Despite the fast-growing interest and demand for TIC in human service sectors, relatively little has been explored about how trauma-informed approaches are applicable and built into services for foreign-born populations in general and for refugees, specifically. Although cultural sensitivity and relevancy is one of the core elements of TIC, it is well known that mainstream social and mental health services in the U.S. are not fully equipped with the level of intercultural capacity to meet unique and diverse needs of refugees and immigrants [13,14,15].

Physiological responses to trauma and subsequent negative effects are considered universal human experiences and yet interpretations and expressions of trauma responses often vary across cultures [16,17]. In fact, a lack of culturally responsive and relevant care has been one of the primary obstacles to serving (im)migrant/refugee communities [18,19]. Behavioral and mental health services in the U.S. do not necessarily account for trauma experiences associated with migration or acculturation, although newly resettled refugees face a unique and distinctive set of challenges in the current care system [17,20].

In addition to cumulative traumatic and adverse experiences during forced migration and displacement, refugee newcomers experience numerous barriers to human services in the host community due to language differences, the culturally alien concept of services (ex. preventive health or mental health treatment), distrust or misinformation about formal services, and lack of transportation, cultural orientation, and information about service systems, to name a few [21,22,23]. Non-western or culturally unique ways of coping and responding to trauma are often unrecognized or unattended to in mainstream systems, so culturally responsive and relevant assessment or service options for refugee populations have yet to be implemented in many service sectors [24,25,26].

A majority of trauma-related programs and models for refugee populations have focused heavily on trauma-specific and trauma-focused interventions, prioritizing clinical treatment and therapeutic modalities rather than organizational- or system-level approaches [27,28,29,30]. For a broader system- or organizational-level improvement to address the aforementioned challenges in service access and disparities in care, it is not only necessary but also inevitable to apply TIC approaches to refugee resettlement programs and related services. Implementation of TIC takes a shift in organizational culture and policy-level changes beyond an individual intervention. However, little has been discussed on the readiness of refugee-serving programs to adopt TIC or the perceived relevancy and acceptance of TIC in refugee resettlement programs. This study aims to explore the current status of the application of TIC approaches in refugee-serving agencies and identify perceived and experienced challenges and opportunities for TIC in the context of refugee resettlement in the U.S.

## 2. Methods

This study adopted a qualitative research method using individual interviews with key refugee-serving professionals in various service sectors. This study was designed as part of a program evaluation with a state-wide refugee health promotion program in Texas, which has hosted the largest number of refugees in the U.S. for the past few years [31]. Refugee newcomers come to Texas from about 30 countries each year, and the number of new arrivals oscillates between 900 and 2500 depending on political atmospheres and administrative decisions at federal and agency levels (See Table 1 for new refugee arrivals to Texas between 2018 and 2020).

### 2.1. Data Collection and Participants

Key community stakeholders in each of five localities were identified and selected by the state refugee health coordinator and the staff of refugee health promotion programs after several meetings with program directors of resettlement agencies and health and mental health professionals in the local community. The first author, as an external consultant, helped refugee health promotion program officers conduct interviews for a needs assessment about culturally responsive trauma-informed care for working with refugees at each resettlement site. The types of service areas included: core programs of refugee resettlement (case managers, employment specialists, cultural orientation instructors), mental health services, medical case workers and/or community health workers, school liaisons/coordinators, healthcare including public health and primary care nurses, community-based organizations serving refugees/immigrants, and refugee community leaders/volunteers. We aimed to recruit 20 service providers per resettlement city, and our final sample included 78 people who participated in individual interviews (See Table 2).

### 2.2. Individual Interviews

Participants from four out of five localities were allowed to choose between a phone interview and an in-person interview, which were held between 2018 and early 2020. For one of the localities, all interviews (*n* = 8) were conducted in 2021 via Zoom due to the COVID-19 pandemic. Interview questions were comprised of five main questions: (1) the relevance of TIC to refugee services; (2) current status of TIC in refugee services, including needs and gaps; (3) challenges in implementing TIC; (4) opportunities and resources for TIC; and (5) suggestions or recommendations for TIC implementation at both agency and community levels. Interviews were conducted by trained social work research assistants at the master’s level as well as the two authors, who are well informed by TIC approaches and principles and added a few probing questions when responses were unclear or too broad. Such probing questions were based on the key elements for the implementation of TIC by SAMHSA [32] as follows:

- Awareness of trauma and trauma impacts (individual staffer)
- Screening or assessment of trauma or trauma-related needs
- Intervention or services to address trauma recovery or promote resilience
- Organizational culture for trauma sensitivity
- Administrative or policy-level support and strategies
- Trauma-informed workforce development (trauma-related training, supervision, etc.)
- Referrals to other resources to meet different trauma-related needs
- Interagency and intra-agency collaboration
- Evaluation or quality control/management for TIC

Each interview took between 30 and 50 min. All participants were aware of TIC and understood TIC approaches and principles, although their experiences with TIC and level of understanding varied. The individual interviews were conducted as part of a program evaluation, and the study protocol was reviewed and exempted by the institutional review board (IRB) of the first author’s institution.

### 2.3. Data Analysis

Each participant permitted audio recordings for transcription and consented for the dissemination of the information after any identifiable information is removed or anonymized. No personal information was obtained, and all interview data were transcribed verbatim by interviewers or a professional transcription company. This study adopted thematic analysis using a template of responses to each question by service providers from different service sectors. Specifically, we used three categories to reveal distinct needs, challenges, and opportunities for TIC in various settings of services. The three types of services included: (1) refugee resettlement services (known as Reception & Placement or R&P programs); (2) mental health services; and (3) others, including community-based organizations (CBOs), schools, psychosocial services, and other forms of services. The template also includes a separate column of overarching themes across all service settings. For cross-checking, the second author conducted initial coding, which was reviewed, recoded, and thematized by the first author. Table 3 presents the final themes of each interview question by service setting.

## 3. Findings

### 3.1. Relevance of TIC to Refugees in Resettlement

Refugee-serving professionals unanimously perceived refugees as survivors of traumatic events who not only have experienced tremendous trauma in the past but also are at a high risk of retraumatization during their adjustment to the new host community. This motivated service providers to indicate strong rationale and interest in TIC in their practice.

#### 3.1.1. Prevention of Retraumatization during Resettlement

Participants were well aware of various resettlement stressors and potential trauma triggers refugee newcomers face, whether apparent or hidden. As a resettlement case manager stated, “traumatization, actually, that is what they will be going through. It does absolutely happen when just moving one person from one country to another where they have no family and lost everything including their support system.” TIC as a service principle was well regarded by providers in all service settings. Several resettlement agency staff mentioned that a key element of TIC should be embedding mental health components, such as screening and prompt referral systems, to the current resettlement programs. As another case manager said,

Including the mental health component of intake assessment [is a part of providing trauma-informed and culturally responsive care]. I think that helps to identify the needs of the client and then helps you tailor to services that you provide so that they include the mental health component and take into consideration the past trauma of that client.

Many respondents mentioned that resettlement services that are focused on “checking off the box” on the list of immediate services are not only insufficient but also distressful and devastating. An employment specialist pointed out,

After 90 days, people have to pay their rent and live on their own. Not knowing anybody to rely on in the community, how can you manage to take care of five children, learn English, learn driving or get a ride, worry about families you left behind, send them some money if you can, and work on a minimum wage job? I can’t imagine how stressful, draining, and even traumatic it can be.

A majority of resettlement agency staff emphasized the critical role of R&P programs in preventing traumatization and retraumatization, indicating how enhanced understanding of trauma may improve the delivery of their services and mitigate frustration when engaging with traumatized clients. Several respondents mentioned, “being in poverty is traumatizing.” A community health worker said,

Our clients are low-income. They are in poverty. They are not making living wages. They have more than likely experienced trauma. There’s one thing to convey to staff that might change or enlighten our services is the real tangible facts. We talk about how to give better services to somebody. It’s regarding this person’s past experience of trauma and the stress that they feel. How does that stress manifest? Is it forgetfulness? I think if we understood the real, tangible, meaningful effects of suffered trauma and living in poverty, then […….] I think that might remove some of the immediate frustrations.

#### 3.1.2. Groundwork for Trauma Recovery

In the meantime, mental health professionals paid more attention to the effect of TIC on therapeutic relationships, trauma recovery, and the de-stigmatization of mental health issues through increased self-awareness and shared understanding of trauma impacts. A mental health professional emphasized, “trauma-informed care is the basis of our work.” Professionals in mainstream human services, such as schools and social services, whose clientele include both refugees and general populations, found TIC a helpful lens to better understand the refugee community and their needs that are shaped or influenced by traumatic experiences. Several participants made an important point about the role of TIC not only in service provision for refugee clients but also for staff to avoid compassion fatigue, secondary trauma, and burn-out, and eventually frequent staff turnovers.

### 3.2. Current Practice

Participants reported two main ways to adopt and deliver TIC in the context of refugee resettlement services: (1) integrating new or additional TIC or mental health components to the existing programs; and (2) prevention of retraumatization through strengthening the current assistance with long-term success in self-sufficiency and integration.

#### 3.2.1. Divide between Tacit Knowledge and Lacking Awareness

There was a clear divide in the current practice of TIC between resettlement programs and mental health services. Most mental health professionals said, “I don’t call it trauma-informed care, but it is part of my job.” To mental health professionals, TIC is considered tacit knowledge required for therapeutic relationships and is embedded in routine practice for mental health care. A clinician in an immigrant family service center described TIC as a guiding principle throughout her support for clients, stating,

My job is to see client facing trauma, so a lot of times psychoeducation component could definitely be helpful because when people come into my office. Refugees or not, or immigrants or asylum seekers, a lot of them are suffering from trauma or are in the stages of crisis. [……] It’s not always the right time to tell someone ‘you might be suffering from the effects of trauma.’ But sometimes when people get out of crisis it can be helpful to just put a name on some of the things that they’re feeling.

On the other hand, respondents from resettlement agencies often stated their practice is not trauma-informed for various reasons. TIC is a relatively new approach to most resettlement agencies, and, thus, lacking awareness or capacity for TIC is commonly reported. For example, one participant stated,

If we have the resources to actually see the signs, we can help this person. They think this is their state of mind, just overlooking that behavior if we are not trained enough to see that this is very typical signs of trauma. If we were trained, then we will help them referred to resources that can help them.

A few participants shared some practical issues and hesitance in providing TIC in resettlement programs due to the concerns about allocating limited time and resources to additional efforts and dispersing the program’s original purpose of self-sufficiency. An R&P staffer argued that current resettlement programs have little room for TIC and emphasized the role of the resettlement program as services to address and get rid of current stressors rather than dealing with past trauma. He stated,

You’re already in crisis and everyone around you reminds you that you need to come up and stand on your feet and move on. Nothing that you’ve been through even matters anymore. What matters is what you have to do from today and on.

Most resettlement staff claimed that there exists a large discrepancy between ideals and realities of the resettlement program. An R&P staffer shared a sense of dissonance and frustration in her efforts to mitigate the gap between the best practice and program requirements, stating,

There is a big disconnect between the idea of our organization having deadlines and a certain amount of time and money that can be spent on people. Then, at the same time, in reality, in order to serve them best, wanting to give them the time that they need to process and make decisions, knowing that you have to be patient with that process, possibly because of trauma. [……] I think one of the more frustrating aspects is having to fulfill the requirements of the job, but at the same time, wanting to give the client autonomy and time. A lot of times, it feels like if a client doesn’t fit into that timeline that it’s almost like the system punishes them for that.

Aspiration for TIC among resettlement staff was often described as frustrating due to the crunched timeline and resources that are too short for adequate support for both clients and service providers themselves.

#### 3.2.2. Growing Needs, Growing Interest

Most participants highly valued TIC, especially when it is also culturally sensitive and responsive, pointing out that TIC is well aligned with their service goals and daily practice manners. A case manager stated,

Whatever we can do to help clients feel heard and feel safe. Especially right now, with this political climate. I think that there is a lot of heightened anxiety, and so prioritizing being able to welcome clients, truly, even to help them feel like humans. And just listening to their needs. I genuinely believe that that [TIC] is the top priority.

As TIC becomes a highly endorsed practice model in diverse service settings, several participants in CBOs and primary care clinics mentioned their agency-wide plan to get trained and deliver TIC. A public health nurse said,

I think it’s important for everyone to at least have a superficial understanding of what to look for and how they can refer to the correct people. And I do know that we have a three-year plan for having all of the centers that provide support to families have a trauma-informed approach.

In particular, professionals in domestic violence shelters/intervention programs and human trafficking victim programs highlighted their trauma-informed services, which increased their partnership and collaboration with refugee-serving agencies towards TIC at the community level.

### 3.3. Challenges in TIC

Respondents shared various challenges in implementing TIC in their service settings despite the growing interest and expressed needs for TIC within their agency and in the community. The main challenges identified by respondents included lack of awareness, knowledge, and skills for TIC, lack of understanding of the refugee resettlement process and culturally responsive practices, and lack of collective capacity for culturally adequate TIC systems.

#### 3.3.1. Lack of Competencies for TIC

Professionals in non-refugee programs identified a lack of training opportunities as a primary gap in their settings. CBOs and health or mental health services reportedly experienced growing opportunities for training on general topics in TIC, and yet few are available for refugee-specific training. A family service provider in a CBO reflected on her needs for refugee-specific TIC training,

Obviously, since our goal in my program is to provide trauma informed service, we had training on trauma, but we didn’t have training specifically about refugees. We would talk about being culturally responsive, but there was no specific training on refugees. Then, I feel like after coming to this [refugee-specific TIC training], I kind of was like thinking like, ‘Gosh, I think families I’ve already been working with, I wish I had a little bit more of this [refugee-specific TIC training] before I even started.’

Capacity issues are also related to the current political climate and reduced refugee programs. As a case manager stated, “it gets back to this issue of capacity that we are just continuously lacking as being short-staffed. I understand to an extent. But there’s so much uncertainty about the future.” This is also commonly faced by non-refugee service sectors as well. A manager of R&P pointed out,

Capacity is, I feel like probably, just not enough. With the current [Trump] administration and climate, less people have been migrating at least in this area. I know there have been budget cuts and things of that nature, so capacity would probably be an issue.

#### 3.3.2. Missing Culturally-Relevant Care

Mental health professionals, in the meantime, shared a different set of challenges, such as lacking information on refugee services and disconnection to other service referrals. A clinician stated,

Having a little bit better understanding of the capacity of the agency could be good. If there was, like, maybe a meeting where all of them had a very firm understanding of what our agency is offering and here’s what we’re trying to refer out. I think that could be good. I’ve noticed some clients getting referred back and forth, which isn’t good.

Discrepancies in capacity across agencies were identified as a major challenge at the community and system levels. Participants commented that providers in non-refugee services were particularly incompetent and even insensitive to refugee needs and background, which creates additional distressing barriers to refugee newcomers. For example, one participant stated,

Sometimes some of the organizations we work with extensively, especially in the government, they don’t really have any training. So, you can do a lot of really great work with the client, where you feel like you’re making a lot of progress, and then they can leave and go to Health and Human Services, and just have a terrible time because the employees there aren’t using the same kinds of principles, so it’s just not matching up in the same way.

#### 3.3.3. Community Capacity and Systems for Culturally Responsive TIC

Most of the challenges described by participants related not necessarily to individual capacity, but also at the community, inter-agency, policy, and structural levels. Resettlement agency employees described a number of challenges related to time, resources, organizational buy-in, policy restrictions, lack of capacity and training, and lack of protocol for TIC. Resettlement staff also expressed concerns about the constraints and barriers to not only TIC but their regular services in general. A supervisor of resettlement caseworkers stated,

I just feel like we’re kind of set up to fail currently in many ways. If we’re not failing the clients in the service we’re providing, we’re failing to be able to fully document it because we just don’t have the time for both and that jeopardizes the future of our ability to defend the funding for it to be given. That takes an emotional toll on all of us. They want to see a certain number of clients served within a fiscal year. They want to see a certain number of units of service provided to those clients within that fiscal year.

Participants in resettlement agencies deplored current resettlement policy for not being informed by refugee experiences, especially refugee trauma, and for creating additional hassles and distress for both their clients and staff. Lacking evidence or understanding of systematic issues was also noted as another challenge. As a case manager shared, “there isn’t institutional support for critical analysis of routine procedure and practice. It’s a system level problem.” Reportedly, both organizational support and policy-structure limited providers’ capacity to adopt and implement TIC in the refugee resettlement program. Some participants in R&P programs perceived TIC as another burdensome task alongside already heavily loaded cases even though the principles and the values of TIC were agreed upon. Many professionals echoed the external challenges, pointing out,

I don’t want to blame external forces all the time because I think there are also organizational changes that can always be made, but I think the biggest barriers are probably rules implemented by other organizations or restrictions on grants or reporting things that feel like they kind of slow down the work we do or even prevent the kind of work that we would like to do.

Transition from early resettlement services to mainstream social services was pointed out as another missing area and the weakest point for delivery of TIC to refugee clients. A CBO program manager shared her hope for extended R&P support that would have facilitated TIC within the resettlement program and beyond.

I think beginning to identify the needs after a year is very helpful for people to make good decision versus when they are at the first month mark. […….] I think it would be helpful [for refugees to receive services for a full year] because your priority is not survival, but your priority is actually planning for your future and recovering.

### 3.4. Opportunities for TIC

As many constraints were derived from external sources, the opportunities for TIC also emerged from both nationally and locally growing discussions on TIC and community-wide initiatives towards TIC.

#### 3.4.1. Growing Interest, Growing Opportunities

Specifically, quite a few mainstream human service agencies, as well as violence survivor programs (e.g., trafficking victim programs, domestic violence shelters, and torture survivor centers) put more efforts for and interest in TIC approaches and training. A bilingual refugee medical coordinator in a community health clinic said,

My agency, maybe like a year ago, they had one workshop about mental illness. At that time, I see people from different community, even from my [refugee] community, they came and they talk about mental illness. They came to know about what is mental illness and these things [TIC]. [……] Our agency did trauma training last year, and I don’t know about this year, but yeah, it would be more I know, we’ll be glad to have more, that type of class for the community.

Non-refugee-specific CBOs also shared an increasing interest in TIC in the community of practice especially among the agencies with national presence and reputation, which helps enhance internal support and buy-in from administration given the top-down chain of communication and command. A CBO staff member said,

But there’s definitely also, in leadership, a strong advocacy for expanding the work that we do. I’m not concerned about it, but I just think that it’s going to take more education on what it means to have trauma-informed approaches on a larger scale and what it means to provide mental health and what are purview is on that.

#### 3.4.2. Disparities in Resources for TIC

Reported opportunities vary greatly across settings and agencies, however. Most of these opportunities arise outside the refugee resettlement program. Some participants in R&P programs reported little exposure to the notion of TIC or trauma-sensitive approaches and have been given little to no resources related to refugee trauma or mental health. An R&P case manager said, “I’ve been working with this program for about five years now, and there was little information that I learned [about TIC].”

Facing a relatively deep divide in capacity and support for TIC between providers in small local agencies and those in large national entities, participants considered it collective responsibility to make refugee services trauma-informed and seamless. Participants across different service settings argued that competency building for TIC can be better facilitated by knowledge transition and exchange among providers through community meetings and interactions with other providers. In fact, in most localities, refugee-serving professionals described holding meetings to discuss difficult cases or share new information and resources. A clinician explained how beneficial such interagency collaborations can be to build TIC, sharing her own experiences. “I am familiar with the other agencies, referring agencies, and a lot of resettlement agencies. I know people at those agencies who I reach back and trust a lot. So, in those regards, it’s good for trauma-informed care.” Interagency dependency is seemingly inevitable to most agencies, but particularly to those with little internal support or capacity, and such collaboration is highly valued and seen as an opportunity for TIC. A community outreach officer in a large city school district explained how they pivot challenges into opportunities for partnerships with other agencies, stating,

I will say we don’t [have resources], particularly our office don’t have the resources [for TIC]. We don’t have the expertise. We do need to gain that, for sure. So, we won’t be able to do it without the help of other agencies, without the help of other resources, and that’s why we won’t be able to do our work without their help.

The school district coordinator added, “a good thing is that we are connected to a lot of resources. It’s like [helping] their own communities, other communities that can help them with that [missing services].” Even when providers did not have knowledge or capacity for TIC as an individual or agency, they managed to support refugee clients through community-wide collaborations.

### 3.5. Recommendations

Participants shared multi-phased steps to deliver culturally relevant TIC to refugee newcomers during the resettlement process.

#### 3.5.1. Training to Enhance Awareness for TIC

An increase in awareness of the impacts of both pre- and post-resettlement traumas and stressors was perceived as either the first step or at least a high priority to implementing TIC in each service setting. Such awareness should be enhanced for not only service providers but also refugee clients, which takes both agency-level and individual efforts. An R&P program manager emphasized the awareness around trauma and mental health as a prerequisite for TIC and stated,

First, training will be for these people’s [providers’] awareness. They should be aware. Then, step by step, for the future, we can have another program for others, for example, for some gathering. [……] Training is important. I think training of personnel, training of clients, training of community leaders, these are the basis for this [TIC].

Participants, regardless of service settings, proposed training as the first step to raise awareness of their clients’ needs and mobilize resources and information for collaborative partnership. In terms of training topics and needed competencies, mental health professionals reported that cross-cultural competency or cultural safety and refugee-specific knowledge should be developed to deliver proper TIC and supplement their existing mental health knowledge and skills. A clinician stated,

For me, I have the background of working in victim services and about trauma-informed care, but they added emphasis on culture, which provided me the opportunity to strengthen my already existing skills. I think bringing this [culturally relevant TIC] to my team and my agency can strengthen my advocates and team members and peers, so they can bring it to the community as front-line impact-changers and creating it, using the same analogy of the pebble and trauma, but for the greater good and changing how our community works with our population.

#### 3.5.2. Resources for Refugee-Specific, Culture-Informed TIC

Providers in all service settings emphasized the importance of refugee-specific contents and protocols tailored to the resettlement context in order to make TIC relevant and responsive to refugee needs. As an R&P case manager stated, “there definitely could be better training and protocol around the best way to broach the topic [of TIC] in a way that won’t just be dismissive and dismissed on either side of the table between client and case manager.” Participants also emphasized that capacity building or training needs to be institutionalized to sustain the efforts. A public health nurse stated,

You have to renew certain trainings and modules every year. I think that the hospital system is getting better at incorporating certain trainings. For instance, it took a very long time but we just got a human trafficking training approved for every new employee. Having something like that with trauma-informed, or in the future maybe refugee trauma in the tool house. I think it takes some time, but that would be the best way to reach everybody.

Moreover, the community’s capacity for TIC was emphasized in the context of the policy structure of the refugee resettlement program. Several respondents in resettlement programs argued that the current resettlement system likely hinders a humanized process of the R&P process, which tends to create multiple issues around an agency’s capacity for TIC. A supervisor of R&P case managers said, “for me, a trauma informed case management would look like a coaching. But our programs are very restrictive or mandated. Very much like X, Y, Z and case closed.” Small refugee programs sitting in a larger service platform that serves general populations often face poor communication and disparities in resource sharing even within the agency. Knowledge transition and inter-program collaboration may promote agencies’ capacity for TIC. As a refugee clinic nurse stated,

Overall, I think we could be more trauma informed. Our behavioral health department is great. Every single one of our therapists has extensive background with training and practice in providing trauma informed therapeutic services. But I don’t know that that extends across the rest of the clinic. So I think that is definitely something where we can improve on. And I think even something as simple as embedding different programs with our clinic that provides opportunity for connectedness to other folks. And that opportunity to really build communities and I think it’s somewhere we’re definitely headed.

#### 3.5.3. Building Partnership for Care Coordination

Respondents unanimously agreed that coordinated care among providers is essential to TIC. Due to the complexity of resettlement challenges, multiple sectors across mental health services and psychosocial programs often need to collaborate to serve a single case. A medical service coordinator said,

I know there’s a consortium of providers and they’ll talk about tougher cases and try to brainstorm on how to help those families. I think the more application-based things are still important because one agency may have a resource that others don’t. Just resource-building, honestly. If I’ve learned anything it’s really about growing those relationships so that you can help the people that you serve. I think the stronger the network, the better the outcome for the client.

Partnerships not only among professionals but also with the refugee community and informal helping sources (e.g., family caregivers and volunteers) were also perceived as crucial to successful TIC. As a CBO staff member shared,

We’re in a position where we’re not able to spend a lot of time. So, for example, there’s like a three hour window that we can do individual work with children. We’re having a hard time finding opportunities to create a community with the families. [……] We set up some events, but then we don’t have our target population show up. So, maybe just different ways of organizing cross-culturally [can work]. Getting other refugees involved, so that they can help us build better relationships, so that the families feel comfortable with us.

Participants suggested that, to have more refugee community members and leaders get involved in mental health programs and successful integration, a community-wide training could provide an effective avenue for relationship building and partnership. For example, a participant stated,

We need the support and help of community, to program. For this purpose, the [refugee] community needs training, especially the leaders, those who are involved in programming and managing, need some training. They should be aware of the importance of this issue [trauma and mental health]. This is really important for them, for their family, they should know about that. Then they can be encouraged by this.

#### 3.5.4. Community Engagement and Inputs for TIC

Community engagement was emphasized repeatedly as another critical element of TIC. As one R&P provider pointed out, staff at refugee resettlement agencies fully understand the importance of “building in some sort of community connections right from the beginning when people arrive to connect people to either people who speak the same language or other mentors in the community.” Many echoed the role of building support systems within the refugee community during early resettlement. In the meantime, there was a raised concern and skepticism about small steps toward TIC without system-level changes. As a case manager in an R&P program shared, “I’m not sure how much you can introduce elements and pieces of it [TIC], but to have a truly encompassing model, I think it would be very difficult to produce one within the current climate and structure of the programs.” Many R&P staff expressed concerns about how changes can be made at the agency level and beyond. In order to create effective solutions, systematic needs assessments and program evaluations seem necessary and yet little evidence has been collected in refugee resettlement agencies.

What would be fruitful is [……] having more opportunities to come to these realizations of how to streamline programs. I do believe that that’s only gonna happen by spending time sitting down and talking to each other more about what’s going on and what we’re doing.

Another R&P staffer elaborated her agency’s plan to fully grasp the current needs and subsequent solutions, which faced obstacles due to lacking competencies for a program evaluation.

There’s so many things we base off of assumption. We don’t have the means to pilot or do a full on focus group study with all of our different client groups. If there was any easier, more accessible way to get that feedback and something that could let us learn more. I think that’s a huge gap from what we do in every single program.

Organization and policy-level changes were highly demanded to create a culture for TIC in the resettlement process. However, frustration and skepticism still lingered as system-level or policy-level changes are seemingly daunting, especially facing adverse sentiment against refugee resettlement in the local community as an R&P staffer said, “I don’t know exactly what it would take at the county level to really put that pressure, and build that momentum to revise that policy.”

## 4. Discussion

Findings of this study highlight TIC as an approach that is relevant and essential to services and community support for refugee populations who face immense acculturative challenges in addition to past traumas. Demand for the application and adoption of TIC was not only from mental health professionals but also from non-mental health service providers who may not directly address or respond to refugee traumas and mental health issues. Given the widespread impacts and consequences of traumas in broad ecological systems of refugee families [33,34], trauma-related needs and resources to facilitate healing and resilience have been highlighted in almost every form of support and various types of human services (e.g., mental health and psychosocial support or MHPSS). Such a wide scope of refugee needs for MHPSS, as well as multiple service systems involved in the refugee resettlement process, entail various challenges and obstacles to TIC. Despite the burgeoning interest and attempt to embrace TIC, there is clear inconsistency and inexperience in TIC in the context of refugee programming, which corroborates previous research on the lack of consensus on culturally competent refugee services [35] and community capacity for appropriate and effective services [36,37,38]. As our findings revealed, disparities in internal supports for TIC across agencies seemingly exacerbate such gaps and discrepancies between client needs and service capacity as well.

This study also sheds light on the role of culturally responsive TIC in community resources and opportunities to bridge the deep divide and substantial gaps between mental health services that are heavily trauma-focused and refugee resettlement services that provide various kinds of psychosocial support other than mental health treatment. Such separation may be necessary for specialization in care and yet lack of communication and coordination between the two tend to engender fragmented care and negligence of comprehensive needs around mental health and wellness. In particular, our findings highlight that trauma-informed care that is culturally responsive and relevant to refugee populations is a vital way to address the chasms between refugee-specific programs and mainstream services. In fact, as a multi-year MHPSS project in the U.S. has shown, culturally sensitive and responsive approaches complement and complete trauma-informed care by filling the gaps in the current refugee resettlement program [27]. In order for TIC to be culturally relevant and responsive to refugee newcomers, there is a need for additional sensitivity and attentiveness to both the individually unique healing process and the collective cultural impacts of trauma consequences in the community (e.g., loss of community and cultural practice, impeded social support systems). Such cultural and contextual relevancy is essential to MHPSS for refugees not only to improve individual service outcomes but also to promote a sense of safety and resilience for social integration of refugee newcomers.

This study has a few limitations to consider when interpreting the findings. First, we interviewed key stakeholders in five different resettlement sites (cities and counties). This provides valuable insight into this important issue with an understudied population. Understanding and awareness of TIC varies among service providers, and as is typical with qualitative research, the responses represented in this analysis may not be representative of experiences in other localities and other resettlement agencies. A multi-state comparison building on the current study could be an avenue for future research. The service sectors and professions of our participants may not be representative, and future studies could be conducted with a more diverse and comprehensive set of community stakeholders, perhaps including interpreters and refugee community leaders. Despite these limitations, this study proposes several significant implications for future adoption and implementation of TIC in refugee resettlement settings.

### Towards Culturally Responsive TIC

In order to build TIC that is effective and appropriate for refugees in resettlement, cultural sensitivity and responsiveness are essential. TIC approaches are known to help enhance and ensure culturally sensitive practice and a sense of safety for those involved in the process [12,39]. However, a scoping review study considering cultural competency in refugee services [35] points out a lack of consistency in the meaning and practice of culturally competent services in refugee-serving organizations. In addition, some empirical studies have revealed that competency building through training of general trauma topics or context-neutral trauma-informed care alone would not guarantee the cultural humility and sensitivity necessary to serve refugees and other culturally diverse groups of clients [40]. In fact, some refugee groups in resettlement experience cultural bereavement, “resulting from loss of social structures, cultural values and self-identity” [41] (p.674). As such, cultural humility and safety should be the foundation of TIC for refugees who likely struggle with acculturative challenges and cultural collisions during resettlement and social integration.

To establish culturally informed TIC, mental health professionals working with refugees should be equipped with the understanding of the refugee’s culture and the meaning of symptoms and symptom expressions in the culture (e.g., cultural concept of distress), learn how these symptoms translate to diagnosis and treatment, and establish an acceptable mode of communication with each patient [17]. Furthermore, implementing a culture-informed approach requires attention to the organizational context and the mental health care system as well as the clinical encounter between the provider and the refugee client [42]. In fact, culturally responsive care is an example of trauma-informed care that emphasizes the strengths of clients. Refugee populations have unique cultural backgrounds, which serve as a source of strength and resiliency. The role of family, view of mental health services, language, and cultural background are all factors that providers should consider when providing mental health support.

This study also suggests that workplace protocols and curricula for TIC should be established in refugee-serving programs and resettlement procedures. Despite the increasing needs and efforts to deliver trauma-informed services to immigrants and refugees [43], service providers face challenges in adopting TIC to their service settings, where the principles of TIC are often perceived abstractly or superficially, failing to engender concrete and pragmatic applications and outcomes that consider the unique resources and challenges of practice contexts. In fact, irrelevant trauma assessment and practice may lead not only to ineffectiveness but also to potential harm for clients [44,45]. For example, events listed in trauma screenings developed for general U.S. populations (such as adverse childhood experiences or ACEs) may fail to capture the collective nature of social suffering and the needs of children who were heavily afflicted by social upheaval and political instability that devastated entire ecological systems. The practice of trauma screening itself may be harmful without organizational readiness and proper internal or referral resources as well [45,46,47]. As such, protocols or training curricula specific to the context of the refugee resettlement program are inevitable for trauma-sensitive care. As Wylie et al. [19] proposed, a transcultural approach may help improve refugee services by providing more competent assessment and screening, building caring environments, and enhancing TIC to be more responsive to refugee needs and mental health issues. In sum, culturally responsive, cross-cultural trauma-informed care provides a framework to build MHPSS programs that are effective and relevant to refugee needs in resettlement.

Finally, partnership building, and community-level and structural changes are critical to successful TIC for refugee and refugee-serving communities. At a systems level, we face policy gaps in refugee resettlement programming. A narrow focus on refugee employment and the expectation that recent refugees quickly be economically self-sufficient ignores their mental health needs. This lack of attention to mental health needs can increase problems and impede social functioning. Furthermore, refugees often lack medical insurance and access to needed services. A lack of partnership and coordination among communities and sectors also limits the quality and timing of services. One solution suggested in the literature is to better understand refugee mental health needs in the context of cultural impacts, such as loss of cultural practice as a source of emotional distress [27], which could help begin to destigmatize mental health issues and provide holistic approaches and services in order to overcome a pathologizing model of mental illness.

There is also a need for culturally responsive interventions and prevention efforts that are grounded to the refugee community and address vulnerability while also promoting resilience. As Miller and colleagues [48] point out, building trusting relationships and advocacy beyond clinical settings and interventions should be in place to promote TIC and make a holistic impact for refugee populations. In order to build culturally responsive trauma-informed systems of care in the refugee resettlement process, collaborative partnerships and collective capacity should be strengthened to build a universal measure for TIC and culturally safe and sensitive organizational policies beyond refugee-serving programs.

## Figures and Tables

**Table 1:** Refugee Arrivals by country of origin in Texas between 2018 and 2020 (Refugee Processing Center, 2021) ^a^.

Country of Origin	2018	2019	2020	Total
**Afghanistan**	52	69	46	167
**Benin**		3		3
**Bhutan**	85			85
**Burma**	443	462	214	1119
**Burundi**	32	6	3	41
**Cameroon**	1	5	1	7
**Central African Republic**		10	1	11
**Colombia**	16	23	6	45
**Cuba**			4	4
**Dem. Rep. Congo**	690	1329	282	2301
**El Salvador**	81	45	44	170
**Eritrea**	107	171	58	336
**Ethiopia**	12	12	8	32
**Guatemala**	2	13	30	45
**Guinea**		2		2
**Honduras**	12	11	20	43
**Iran**	10	33	38	81
**Iraq**	25	94	57	176
**Ivory Coast**	4	8		12
**Nepal**	3	1		4
**Pakistan**	37	17	5	59
**Palestine**		5	3	8
**Republic of South Sudan**			5	5
**Rwanda**	23	18		41
**Senegal**	1			1
**Somalia**	9	18	1	28
**Sri Lanka (Ceylon)**	1	2		3
**Sudan**	13	36	13	62
**Syria**	1	39		40
**Turkey**		1		1
**Uganda**	3			3
**Vietnam**	25	24	8	57
**Zimbabwe**	8	1		9
**Total**	**1696**	**2458**	**902**	**5056**

^a^ Individual refugee arrival data between 2018 and 2020 were combined and reorganized by country of origin for this paper.

**Table 2:** Individual Interview Participant Demographics: (Total N = 78).

Variable	Frequency (%)	*M* (*SD*)
Gender		
Female	49 (68.1%)	
Male	23 (31.9%)	
Age (in years)		38.51 (11.69)
Prior experience working with refugees		
Yes	70 (89.7%)	
No	8 (10.3%)	
Time working with refugees (in years)		5.89 (5.58)
Profession ^a^		
Mental health service provider	13 (12.9%)	
Healthcare provider	8 (7.9%)	
Refugee resettlement services	26 (25.7%)	
Interpretation	12 (11.9%)	
Medical case worker, community health worker	8 (7.9%)	
School coordinators, staff in school setting	10 (9.9%)	
Refugee program supervisor	4 (3.9%)	
Refugee community leader/volunteer	8 (7.9%)	
Community-based organizations	8 (7.9%)	
Other ^b^	4 (3.9%)	

^a^ Adds up to more than 100% because participants could select multiple options; ^b^ Others include legal services and human trafficking victims programs.

**Table 3:** Thematic analysis by and across service sectors.

	Refugee Resettlement Services (i.g., R&P Programs)	Mental Health Services	Others (e.g., CBOs, Schools, Social Services)	Cross-Sectional Themes
**Relevance of TIC**	Trauma awareness for better stress management and adjustment Useful tools for dealing with resettlement stressors TIC for early detection of mental health needs TIC to supplement and/or complete R&P programs TIC for preventing re-traumatization Providing culturally relevant care is TIC	TIC as a key element of healing TIC to increase self-awareness of symptoms TIC to build healthy, effective therapeutic relationship TIC to promote client resilience	TIC needed for identification of needs TIC as a lens to understand the refugee community Refugees as the most needed client group for TIC	TIC needed for both staff and refugees (e.g., prevention of burnout, self-care practice) TIC as a way to destigmatize trauma-related issues TIC to build trust and safety
**Current status**	No room or no time for TIC Lacking awareness and capacity for TIC Emerging interest in TIC Provision of culturally sensitive care as TIC No additional resources or no new actions for TIC	Tacit knowledge on TIC TIC being embedded to current services Low access to TIC among refugees Basic services missing for refugees TIC being placed under different names or approaches (e.g., strength-based approach, compassionate care, patient empowerment, etc.)	TIC being practiced mostly outside refugee programs In the process of expanding TIC to refugees TIC implemented for specific subgroups (e.g., human trafficking victims)	Some elements of TIC being implemented in current services Wide variances in readiness and capacity for TIC among agencies Emerging interest and voices for TIC for refugees
**Challenges**	Lack of training for TIC Lacking administrative buy-ins Unrealistic R&P timeline Discontinuity of services between resettlement agencies and the mainstream services (including mental health care) Lack of systematic assessment of the program (e.g., lack of needs assessment, program evaluations, and evidence) Policy obstacles (insurances, types of services being provided, R&P services) Political sentiment of anti-refugees Lack of protocol for TIC relevant to refugees TIC and mental health deprioritized Program’s interest in output not in outcome (checking boxes of given services) Lack of information (e.g., proper referrals, competent providers, etc.) Budget cut and program insecurity	Cultural relevancy and sensitivity of TIC Lack of communication, outreach, and partnership—with refugee communities Lack of communication and partnership—with other service providers Lack of cultural competency or humility among clinicians Restrictive schedules, duties, and/or limited time for inter-agency collaborations Language barriers or lack of competencies for working with interpreters No/little organizational buy-ins for community outreach	Lack of TIC protocol, resources and/or training in the context of refugee services Chasm between mainstream services and resettlement programs Lack of competencies or programs that meet refugee-specific needs (e.g., transportation, interpretation, cultural humility/safety) Anti-immigrant/refugee sentiment of the community Lack of communication and interactions with internal resources	Wide variances in capacity for TIC across agencies and service fields Lack of communication between agencies and within agency Limited referral information and resources Lack of support or platforms for collaboration and coordinated care Limited interagency accountability
**Opportunities**	Interagency meetings (infrequent but steady) Sparse chances of training Increase in exposure to trauma-related terms Staff’s commitment	Internal support/resources for TIC Expertise in mental health	External supports and community initiatives of TIC (e.g., school-wide TIC) Agency’s internal resources (e.g., more training opportunities for general topics in TIC)	Emerging discussions on TIC both inside and outside refugee programs
**Recommendations**	Having key champions at administrative or managerial levels Policy-level changes are prerequisite for TIC in resettlement Regular training on TIC (for new and existing staff) Education on trauma and its impacts to refugees (e.g., cultural orientation) Creating a protocol for TIC relevant to refugee services	Cultural sensitivity as a key to successful TIC Collaboration with the refugee community Working better with interpreters TIC training for interpreters and other collaborators	Collaboration among various service sectors and agencies Boosting communication within an agency (to utilize internal expertise and resources for TIC) Community-level education or training on TIC	Both agency-level and community-level (i.e., interagency) training for TIC Exchanging knowledge and resources across service sectors through collaborative partnership Adopting TIC as community building and advocacy tool Protocols for refugee-specific TIC

## Data Availability

The data are not publicly available due to privacy or ethical restrictions.
